# Introducing career skills for dental students as an undergraduate course at the University of Szeged, Hungary

**DOI:** 10.1186/s12909-020-1981-4

**Published:** 2020-03-06

**Authors:** Réka Magdolna Szabó, Joan Mary Davis, Márk Antal

**Affiliations:** 1 Department of Health Economics, University of Szeged, Faculty of Medicine, Tisza Lajos körút 109, Szeged, 6725 Hungary; 2 Missouri School of Dentistry & Oral Health, A.T. Still University, 1500 Park, St Louis, Missouri, 63501 USA; 3 Department of Esthetic and Operative Dentistry, University of Szeged, Faculty of Dentistry, Tisza Lajos körút 64, Szeged, 6720 Hungary

**Keywords:** Dentistry, Career development, Problem-based learning, Curriculum

## Abstract

**Background:**

In the last three decades there is a growing recognition in the dental profession that dental education must go beyond teaching the technicalities of dentistry and include professionalism and communication skills that the future dentist may need. Such skills are best taught in a student-centered way. Literature suggests that student-centered elements are difficult to introduce in traditional, teacher-centered curricula. This is especially true in post-communist countries where higher education was under strict state control for decades. The aim of the piece of research presented here was to investigate how difficult it is to introduce a student-centered career skills course in a traditionally teacher-centered dental curriculum.

**Methods:**

Considering the needs of our final-year dental students and Super’s model of career development, we created an undergraduate curricular career skills course running for two semesters in two languages (Hungarian and English). The primary aim of the course is to help students with their career expectations and develop their identity as a professional workforce. The secondary aim is to teach skills that students can use when applying for a job. At the end of the semesters, we assessed our students’ satisfaction with various aspects of the course by using a questionnaire. Results were analyzed item-wise and according to the main aspects of the course (i.e., groups of items organized around a particular aspect).

**Results:**

General satisfaction with the course was high, and practical skills training (such as CV and motivation letter writing) got the highest scores. From the answers it appears that the students were the least comfortable with having to deal with their personal values and preferences.

**Conclusions:**

While it is common for universities to offer various forms of career intervention, to our knowledge, no other university offers a curricular career skills course specifically for dental students. Our student-centered course designed in a problem-based learning framework worked even in a traditionally teacher-centered educational environment, where university students are rarely encouraged to be active participants in courses. By sharing our experience, we would like to encourage our fellow dental educators working in similar environments to devise and offer such courses.

## Background

The undergraduate dental curriculum “should prepare graduates to enter practice” [1]. What it means depends on what is considered practice-related by a given curriculum. Literature and most importantly experience tell us, though, that dentistry is a particularly demanding profession that requires a wide range of non-clinical skills. As Myers and Myers summarized, ‘It’s difficult being a dentist’ [2]. This is partly because of the plethora of health hazards [3–7], but also because of the difficulties in balancing work and family [8] and the fact that dentists need to be competent also in non-medical aspects of their practice, such as financial planning, addressing legal issues, personal time management, or practice organization. Studies have found that both dentists and dental students tend to find such practical matters challenging [9]. It should come as no surprise that dentists are exposed to high levels of stress, and none of the specialties seems to be an exception [10]. Constant stress paves the way for alcohol problems [11], burnout [12], and, in the most severe cases, suicide [13]. Accordingly, dental schools around the world have started to address these issues in their curricula. Dealing with the non-strictly professional challenges of dentistry, however, requires certain ‘soft’ skills (e.g. communication, knowledge of one’s self, etc.) that cannot be taught in a teacher-centered framework.

Introduction of a student-centered approach, however, can be difficult. Resistance to change in higher education is a well-known phenomenon [14], toward which both faculty and students contribute. Analyzing the reasons on the faculty side, Brickner differentiated between first-order and second-order barriers to change [15]. The former category includes extrinsic factors, such as insufficient time to plan instruction or inadequate support, and the latter includes intrinsic ones, like beliefs about teaching and learning, strong adherence to established classroom practices or, simply, unwillingness to change [16, 17]. Students, on the other hand, are often socialized to be and used to being passive recipients of academic information [18]. Jain et al. [19] point out that ‘teaching in most Asian countries is still dominated by teacher-centered classrooms, in which students passively receive information from the teacher and internalize it through memorization’. The authors add that while such students may find an active role uncomfortable, they often find the passive way of learning unsatisfactory. In summary, the wish for change may well be present together with resistance to it.

Several dental curricula, however, still prepare graduates predominantly in the technical sense, while crucial aspects of dentistry as a job are paid only cursory attention. Cerych, summarizing the situation right after the fall of the Soviet Union, pointed out that the rigid centralization and almost total political control of higher education turned resistance to change into the normal state of affairs in the occupied Central European countries. As the author laconically remarks, “…the majority of people in higher education in Central Europe do not know how to do things differently. They are naturally aware of, and even adhere to, general concepts such as university autonomy or freedom of teaching, but too often these are merely postulates without practical consequences. Worse, they may be postulates leading to problematic simplifications […] a new curriculum meaning just getting rid of the ideological constraints, without moving toward new structures of knowledge.” Then he goes on to cite a Czechoslovak specialist: “One of the greatest problems is that the majority of teachers are not interested in curriculum change” [14]. Almost 30 years after the democratic turn, the situation looks quite similar, at least in Hungary. What conserved this attitude of resistance and the old structures as a consequence is a complex issue, the discussion of which is clearly beyond the scope of this paper. Still, its pervasive presence is a fact. At the same time, the literature of medical education and international experience suggest that the introduction of student-centered elements is imperative to enhance the quality of dental education [20–22].

What we present here is a curricular career intervention course for senior (5th -year) students that we introduced into the undergraduate dental curriculum at the Faculty of Dentistry, University of Szeged, Hungary in the academic year 2017/2018. The course was approved by the Faculty Council (Decision No. 4/2017 (2017.01.31.)).

Given the described educational atmosphere, we initially assessed our students’ opinion about this more student-centered way of teaching and the contents of the course with the help of a questionnaire (see below). Our hypothesis was that the course would be generally welcome by students, and the practical aspects of it would be the most popular. We also hypothesized that other aspects, especially having to talk about one’s strengths and weaknesses, would be less appreciated, as they are unusual for a university course and generally alien from the educational culture our students were socialized in.

### The educational context

In Hungary, dentistry is a 10-semester master’s program. The four universities that offer dentistry (Budapest, Szeged, Debrecen, and Pécs) have different curricula, but these are all organized around the same main phases or modules: foundations (basic medical sciences and social sciences related to medicine, such as ethics and medical psychology), preclinical studies (laboratory work and the basics of the most frequent dental interventions), and clinical studies (treatment of patients under supervision). Students obtain their DMD degree after the successful completion of written and oral state board examination. At this point, they are also immediately licensed and registered as general dental practitioners by the National Public Health and Medical Officer Service. Thus, it is possible for the newly graduated dentist to start practice immediately. Those who wish to keep their right to practice must participate in continuing education. Other options include specialty training (3–4 years at university clinics or licensed private practices), PhD studies (4 years), or any job that requires a dental degree. The path to a career in dental education usually starts with specialty training and typically continues with PhD studies, as a PhD in clinical medicine is a requirement for promotion beyond assistant professor. Dental educators in the clinical studies stage of the undergraduate curriculum are always practicing dentists.

Career counseling or support is not part of the curriculum at any level. Such activities are mostly organized in an extracurricular fashion. The University of Szeged, for instance, has a Career Office, which organizes job fairs and provides an online advertising surface for firms offering jobs, but - unfortunately - career counseling is not part of the office’s portfolio. Furthermore, this service has no profession-specific elements, so the specific needs of dental students are not addressed at all.

It was in this context that we designed and introduced the course delineated here. Our initiative was that as dental educators, we found that our senior students were often at a loss when faced with the reality of having to choose a career and enter the job market. Some of them did not have any idea about what careers were possible with a dental degree apart from chairside work, but we often met students who had not even thought about their career before the end of their studies. Our goal was to help these students at this critical point. Here we describe the course and our initial experiences with it, with an emphasis on student feedback.

## Methods

### Aims of the course

In terms of career development, the course sets out aims at multiple levels. To formulate our aims, we used Donald Super’s theory of career development as a framework [23, 24]. In Super’s career lifespan, university students fall into the specification (18 to 21 years of age) and implementation (22 to 24 years of age) substages of the exploration stage (15 to 24 years of age). The goals linked to these substages are career planning, training for the specific goals, and career initiation. Strictly speaking, fifth year dental students (aged 22–23 years) fall into the implementation substage. However, Super’s model of career development as a model of linear development built of consecutive stages could work only in a system that actively supports the accomplishment of every stage. The real situation is not so clear-cut. Hur and colleagues demonstrated how different medical students can be in terms of how ready they are for a career [25]. We had similar experiences. Therefore, we decided to add aims to address the entire exploration stage, including also the crystallization substage (15 to 17 years in the original model). The main reason for including that early substage was that dentistry opens the way toward various career tracks (as mentioned above), of which chairside work is but one. We assumed that our students did not have that information in high school, when they went through their “first” crystallization stage, and we wished to give them the chance to briefly revisit that substage with the new information in mind. This is of vital importance, as the goal of this substage is the self-assessment of one’s needs, values, competencies, and opportunities. Without that, no sound career decision is possible.

The primary aim of the course is to help students with their career expectations and develop their identity as a professional workforce. The secondary aim is to teach skills that students can use when applying for a job. After completing this course, students should be able to write a CV and motivation letter, prepare their own professional portfolio, and assess if a potential workplace meets their character, goals, and expectations. The rationale for this is twofold: first, it has been documented that organizing background materials, practicing answers, and doing one’s own research on potential employers could improve the outcomes of a job interview [26]. Second, activities related to the secondary aim also contribute to the primary aim. Detailed study goals are presented in Table 1.

**Table 1:** Course structure

Addressed career development substage	Topic/Activity	Study goal	Allocated time	Course phase
CR	Opportunities as a fresh graduate	The student knows about his or her opportunities as a fresh graduate (including dental work)	1 h	TP/C
CR	Career options as a dentist	The student knows about his or her opportunities specifically linked to the dental degree	1 h	TP/C
CR	Assessment of personal character traits, strengths and weaknesses	The student has a fundamental career-related concept of himself or herself as a person	2 h	TP/C
IMP	Study sample CVs to demonstrate main points of CV writing	The student knows the formal and stylistic requirements of a professionally written CV	2 h	TP/C
SP	Coaching in career planning (optional, by appointment)	The student has personalized feedback on his or her actual career dilemmas	1 h/student	TP/C
IMP	Preparation of own CVs (extra-class)	The student has a formally and content-wise correct CV	NA	PP
IMP	Motivation letter and professional portfolio samples	The student knows what (not) to include in a motivation letter and portfolio	1 h	PP
IMP	Preparation of own motivation letter (extra-class)	The student has a motivation letter	NA	PP
IMP	Evaluation of CVs in group, suggestions, corrections	The student can critically analyze a CV and use this knowledge to enhance his or her own CV	1 h	PP
IMP	Evaluation of motivation letter in group, suggestions, corrections	The student can critically analyze motivation letter and use this knowledge to enhance his or her own motivation letter	1 h	PP
CR/SP	Making career decisions	The student can identify short- and long-term goals (vs. consequences) and think consciously about a career decision	1 h	PP
SP	Establishment of short- and long- term goals	The student can set well-defined career goals and expectations for himself or herself	2 h	PP
IMP	Simulated interview (individual)	The student has prepared for a job interview-like situation and tried what it feels like to be interviewed for a job	4 h	IP
SP	Assessment of possible workplaces/jobs	The student knows how to gather information about a potential workplace/position and how to assess that information	1 h	IP
IMP	Job fair	The student meets real employers, has a chance to apply for jobs	4 h	IP

Legend- TP/C: theoretical preparation/contemplation; PP: practical preparation; TO: task to be completed outside the class IP: implementation practice; CR: crystallization; SP: specification; IMP: implementation (Career development substages from Super’s model, see Methods)

### Course design

The course consists of three consecutive phases, and the various activities included address the different substages in Super’s exploration stage, as described above. The phases and tasks related to the substages are shown in Table 1.

The first phase consists of introductory lectures about job options and opportunities available for the newly graduated dentist. These lectures provide detailed information about specialization as well as about working as a private dentist versus a state employee (or a combination of the two). Emphasis is placed on the personal attitudes, skills, and abilities that students need to find their place in the settings discussed above. Although the primary goal of these lectures is to familiarize students with local (Hungarian) characteristics, an overview of key differences between EU member states and some overseas countries, such as the United States and Australia is also provided. While focusing on a well-defined topic, these lectures are also interactive and flexible in many ways. First, the students are actively involved, they can ask tquestions any time, or even change the direction of the lecture within the limits of the topic. Second, students have the chance to ask the lecturer to incorporate information about specific settings, countries, etc. of interest into the lecture that follows. This way, each student group can fine-tune the lectures to their own needs and interests. Third, dental professionals are regularly invited as guests (either in person or via Skype), so participants can gain more personal experience and impressions. The general aim of this theoretical preparatory phase is to support “re-crystallization”, that is, to help students set realistic career expectations by providing adequate information and role models.

The second phase, practical preparation, focuses on CV and motivation letter writing as key skills necessary for a successful job application. The characteristics of the genres are explained, and samples are provided. As an extra-class assignment, students must prepare their own CV and motivation letter. The instructor is actively involved in the writing process by giving feedback on the draft versions and recommending changes if necessary. The primary aim of this phase is not to teach these genres but to provide a chance for students to think about their character and goals and to go through their strengths and weaknesses. The resulting “inventory” not only helps the individual student to think about what to communicate when first applying for a job, but also to identify an apparently optimal starting point for his/her career. As the course is announced for students in their final year, CVs and motivation letters prepared here can directly be used.

In the third phase, students finalize their CV and motivation letter, and individual mock job interviews are video-recorded. The recordings are then evaluated by students and teachers together. We chose interview simulation because interview is the most frequently used and most highly weighted employee selection method, and evidence suggests that interview performance can be efficiently improved via coaching and feedback [27–29]. In the same phase, the course culminates in a job fair, which is a real-life, semi-formal encounter between the participants of the course and various local employers. Employers are invited to register if they are interested in employing newly graduated dentists. The event is organized at the end of the semester, usually at a popular local off-university meeting place, so personal encounters between students and the representatives of employers can take place in a neutral environment. The representatives of the employers have the CVs and motivation letters of the students, and they can talk to those whose introduction they like. The session has no strict rules. The students may also initiate conversation with the employers of their choice. Such a session lasts 3 to 5 h and is best described as a social event with a well-defined purpose.

An optional, individual, sixty-minute coaching session is also part of the course. This is done by appointment. Although this session is optional, every participant has taken the opportunity so far.

The course is taught by a two-person team: an experienced dental educator (who is also a practicing dentist, MA) and a health and marketing communication specialist (RAS).

Student evaluation is based on participation, class activity, and the quality of the prepared materials. A five-point grade system is used, where 1 means failed and 5 means excellent.

The number of participants is limited to 15 students/semester/language (the course is held in English and in Hungarian, see below).

Finally, the course is optional but with a credit value; completion of the course counts toward the completion of dental studies.

**Fig. 1:**
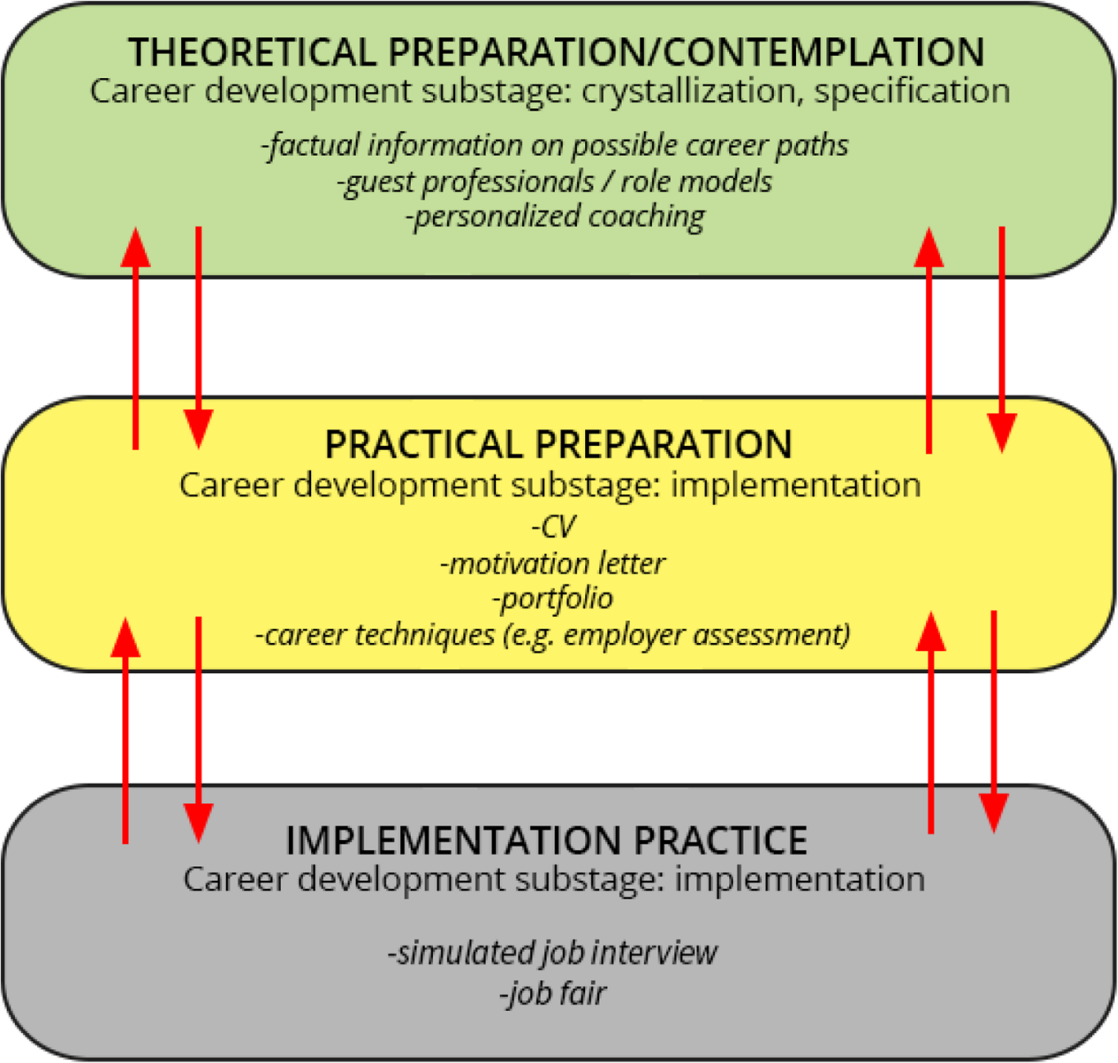
Components of the course and their interaction. The theoretical preparation/contemplation phase supports the crystallization and specification substages. By offering factual information, showing models and reflecting on the student as a professional who is about to enter the job market, the phase aims at helping students find a job they can identify with. The practical preparation and implementation practice phases both support the implementation substage. The motivation letter and CV have dual purpose: they help the student identify the path he or she wishes to take, but later these can also be used for actual job applications. The mock job interview (with feedback) help students reflect on their self-promotion skills in an interview situation and offers starting points for enhancement if necessary. The portfolio is a material means of self-promotion that the students can use for job application, just like the CV and motivation letter prepared in the course. The arrows indicate that the phases can influence each other in both ways

The course components and their contribution to the aims of the course are summarized in Fig. 1.

### Participants

So far two semesters have been completed and 39 students have finished the course (12 men and 27 women). The mean age of the participants was 24.6 (±2.3) years. All students were in their fifth (final) year at the Faculty of Dentistry, University of Szeged. As the Faculty offers dental education in two languages (Hungarian and English), this course was also held in these two languages. Of the 39 students, 28 attended the Hungarian course (mean age: 23.9 ± 1.3 years) and 11 attended the English course (mean age: 26.4 ± 3.2 years). Participants of the English course came from 7 different countries (both EU and non-EU countries).

### Student feedback

A short anonymous questionnaire about the course and its methodology was administered to all participants to assess the reception of the course. The questionnaire was approved by the Institutional Review Board at the University of Szeged. The questionnaire consisted of 20 items, of which the first two were demographic in nature (gender and age). The rest were statements, which the students had to evaluate on a 5-point Likert scale (1- strongly disagree, 2- disagree, 3- neither agree nor disagree, 4- agree, and 5- strongly agree). Two of the 18 statements were negative controls to check for the validity of answers. The statements aimed to assess five main aspects: general impression, satisfaction with the course (2 items); personal development (4 items); CV/motivation letter/portfolio (5 items); career development techniques (e.g., assessment of potential employers, 4 items); job fair (3 items). Each statement was characterized by a mean score (with standard deviation).

**Fig. 2:**
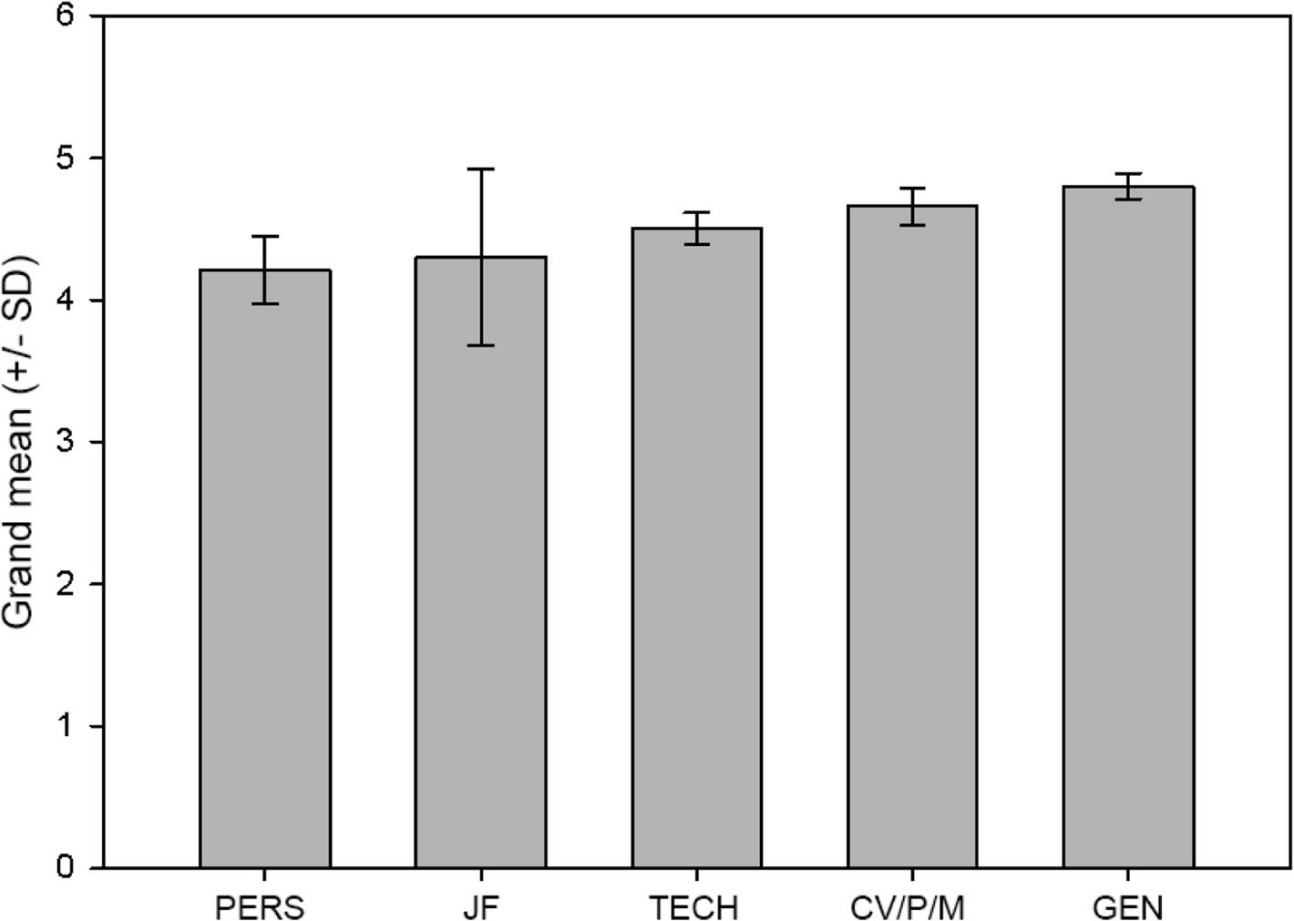
Results of the aspect-wise analysis. Bars represent grand means, error bars represent ±SD. The abbreviations are the same as in Table 2

Mean scores were calculated as the simple arithmetic mean of the individual scores given by each student (1 to 5). A higher mean, therefore, represents higher average agreement with the given statement (except for the negative controls). An initial ANOVA indicated no significant difference between Hungarian and English-speaking students in any of the items (at *p* < 0.05), therefore the two groups were analyzed together. This method of data analysis was preferred, as Hungarian students predominated in our sample (~ 72%), which made a separate analysis meaningless. The item-wise summary of the results is presented in Table 2. Results were also evaluated by question sets (i.e., regarding the five main aspects described above) by calculating the grand mean of all items. Results of the aspect-wise analysis are shown in Fig. 2. Calculations were done in SPSS 21.0 (IBM, USA).

**Table 2:** Student feedback on the course

STATEMENT	Mean	SD +/−	Group
1. Guidance on how to create a professionally written CV will help me get the job I want.	4.85	.366	CV/P/M
2. The assessment of my personal character traits, strengths and weaknesses helped me to clarify what career fits me best.	4.13	.767	PERS
3. I found it uncomfortable or embarrassing to explore my personal characteristics, strengths and weaknesses.	1.77	.959	PERS*
4. Learning about what jobs I can have and what they mean in terms of career made me more confident about looking for a job.	4.46	.600	TECH
5. Individualized career advice received in this course did help me plan my career.	4.62	.747	TECH
6. Guidance on how to assess a potential employer/position helped me create short and long-term goals.	4.41	.751	TECH
7. Studying sample CVs helped me create a CV that accurately reflects the skills I can bring to a job.	4.64	.668	CV/P/M
8. The evaluation of a professionally made portfolio sample gave me a clear idea of how to develop my own professional portfolio.	4.49	.601	CV/P/M
9. Guidance on how to assess a potential employer/position did not me create short and long-term goals.	1.31	.569	TECH*
10. The feedback I received following the simulated interview allowed me to identify areas that need improvement before an actual interview.	4.03	1.197	PERS
11. The interaction, suggestions and feedback received during the group discussion helped me improve my own CV.	4.49	.644	PERS
12. The feedback I received on my motivation letter gave me clear guidance on how to improve it.	4.62	.544	CV/P/M
13. The feedback I received on the materials I created in this course made me more confident about applying for an actual job.	4.69	.521	CV/P/M
14. I think that the Job Fair has been a useful experience in terms of finding my future workplace.	4.64	.628	JF
15. The Job Fair gave me an increased sense of self-confidence for future job interviews.	4.46	.720	JF
16. I found workplaces at the Job Fair that I can imagine as my first workplace after graduation.	3.77	1.459	JF
17. I feel that the personalized/interactive format was a more effective way to learn in this course than a lecture format would have been.	4.82	.451	GEN
18. Overall, this course gave me the knowledge, confidence, and motivation to seek employment after graduation.	4.69	.731	GEN

Legend- Groups of questions: CV/P/M: CV, portfolio, motivation letter; PERS: personality and self-knowledge; TECH: career-related techniques; JF: Job fair; GEN: general impressions. *: negative control question

As the course is new, it is difficult to assess whether the course influences success in finding a job or job satisfaction. Therefore, we focus on student feedback on the course itself, which is an important indicator of how effective the course is, and how well it fits into the curriculum. Obviously, if students find a course useful, interesting, and well-constructed, the efficacy of that course in terms of knowledge transfer increases.

Our hypothesis was that the course would be generally welcome by students (high mean scores on positive items).

## Results

The results are shown in Table 2 and Fig. 2. As for the item-wise results (Table 2), most statements received a mean score above 4 (agree or strongly agree), which indicates a high level of general satisfaction with the course. These results confirmed our main hypothesis. Standard deviations were low with two notable exceptions, item #10 (4.03 ± 1.197) and item # 16 (3.77 ± 1.459). These items ask the respondent about the perceived effect of feedback on the simulated interview and if he/she has found a potential employer during the job fair, respectively.

Negative statements #3 and #9 received a mean score below 2, indicating that participants did not find having to think and talk about their own personality excessively uncomfortable, and they did not consider knowledge about how to assess a potential employer useless (the latter was control question for item #6). The results, therefore, did not confirm our hypothesis regarding the personal/psychological aspect, but standard deviation of the mean of item #3 (1.77) was almost 1.0, which is the third highest standard deviation.

Results of the aspect-wise analysis are presented in Fig. 2 in an increasing order of grand means. All studied aspects scored between 4 and 5, but an order is clear. The general satisfaction aspect got the highest grand mean, which again supports our main hypothesis. As for the particular aspects, practical matters, such as CV writing, composition of a motivation letter, and other career techniques earned the second and third highest grand means. Job fair ended up fourth (with high standard deviation), and activities/tasks related to personal development were associated with the lowest grand means.

## Discussion

By developing a career skills course for dental students, we wished to address a simple but important problem: in our teacher- and academic knowledge-centered system of dental education, the newly graduated dentist often finds that at the university, he/she learned the entire profession, but got little knowledge on how to reflect on himself/herself as a professional or as someone who is about to start a career. The lack of career skills and a concept of oneself within the profession may lead to mistaken career choices and secondary job dissatisfaction, which ultimately leads to burnout, and the individual gets stuck in a situation that leads to a severe deterioration of quality of life. This is not unique to dentistry, but - as outlined in the introduction - dentistry is a particularly stressful profession. Unfortunately, this is true not only for chairside work; being a dental teacher [30] or a dental leader [31] can be the source of just as much stress. We propose that being conscious about one’s professional self, making conscious career choices, and having a sound knowledge of one’s possibilities with a degree in dentistry (i.e., mobility) can serve as important coping resources.

We considered it to be of crucial importance to introduce this course as curricular with a credit value. There are two main reasons for this. First - even if it is not a mandatory course -, if a course is curricular, it carries the message that the faculty considers its contents related to the profession. Second, a credit value means that the student is rewarded for participation also in terms of progress in his/her studies. This latter aspect is important because students in our dental school are on an especially tight schedule in their final year, and they carefully assess the cost- benefit ratio of any subject they take. Therefore, it is possible that they do not take an optional course they would otherwise consider interesting or useful. With this format (optional curricular course for credit), we reached an above-average number of participants for a final-year optional course in the Hungarian group and average participation in the English-speaking group. The reason for the latter is probably that only a fraction of our English-speaking students are interested in Hungarian career options, those who wish to stay in Hungary for practice or specialization after graduation. This observation tells us that the English part of the course needs to be fine-tuned so that it can address a wider range of our English-speaking students.

Our hypotheses, described above, were mostly supported by the results. Students were highly satisfied with the course, and practical aspects (CV, motivation letter, etc.) were among the highest-rated ones. According to the item-wise analysis, the most appreciated features of the course were its interactivit (item #17; 4.82 ± .451) and the chance to learn how to write a CV properly (item#1; 4.85 ± .366). Of the five items obtained the highest scores, three were related to practical matters and two assessed general satisfaction. The item received the lowest score was #16 (“*I found workplaces at the Job Fair that I can imagine as my first workplace after graduation.”*). This came as no surprise, as the job fair offers a rather narrow, strictly local sample of employment possibilities. Some may find a job here, but it is more a training site where students can practice meeting employers without the usual real-life risks. Apart from this, the job fair scored high, as item #14 (*“I think that the Job Fair has been a useful experience in terms of finding my future workplace.”*) received a score of 4.64 ± .628. Items related to personal characteristics/personal growth, however, tended to obtain lower scores. This result might reflect the fact that Hungarian students (or students studying in Hungarian higher education) are not used to being given the chance to look at themselves as significant actors in a university course, as mentioned in the introduction. Therefore, the scores do not reflect dissatisfaction, but rather the unusualness of the situation. This result may also reflect that it is more difficult to assess the use of self-knowledge and various psychological skills than that of a CV or a motivation letter. A further possibility is that statements like “*The assessment of my personal character traits, strengths and weaknesses helped me to clarify what career fits me best.”* (item #2) are just too difficult to score shortly after the course, as such changes probably take more time. In this sense, the results may simply reflect the inaccurate wording of the corresponding item, which prompts us to revise these items before further use. The results of the aspect-wise analysis (Fig. 2) corroborate these findings.

Most importantly, our student-centered course worked surprisingly well in a traditionally teacher-centered educational environment, where university students are rarely encouraged to be active participants in courses. This is in contrast with the results of studies describing the difficulties of introducing student-centered elements in teacher-centered curricula [32, 33]. We propose that the success of the course can be traced back to a few key factors. The first of these is the very subject of the course and its place in the curriculum. It is known that the opportunity to formulate meaningful personal learning objectives is a way to engage students in deep learning processes [16, 34, 35]. To senior students, starting a career is indeed an immediate and real personal goal, and working toward that goal is a logical and meaningful objective. Students of the lower grades would probably have found the course less engaging. The second factor is obviously the credit value of the course, through which the course did not only provide the students with important and personally meaningful skills, but also helped them toward finishing their studies. Third, the course was designed and offered by faculty members who were dedicated to the idea of student-centered education, which excluded the problem of faculty reluctance.

It is certainly a weakness of this study that the present instruments allow the assessment of short-term effects and impressions only. For a more accurate and less subjective assessment, we are planning add tests to measure various aspects of career readiness, like Hur and colleagues did in their study of medical students [25]. Finally, we cannot determine at the moment how lasting the effect of the course is. To answer that question, follow-up needs to be organized.

It is certainly a weakness of this study that the present instruments allow the assessment of short-term effects and impressions only. For a more accurate and less subjective assessment, we are planning add tests to measure various aspects of career readiness, like Hur and colleagues did in their study of medical students [25]. Finally, we cannot determine at the moment how lasting the effect of the course is. To answer that question, follow-up needs to be organized.

## Conclusions

While it is quite common for universities to offer career skills trainings for undergraduate students, to our knowledge, no other university offers a curricular course of this kind, tailored especially to the needs of dental students. To our knowledge, the closest example is embedded counseling model described by Adams [36], but that model is more focused on students’ problems while still at school than their future career.

By sharing our experience, we would like to highlight benefits of formal instruction in career planning skills. Our experience suggests that such a course can be implemented in a teacher-centered educational environment with relative ease, provided that a few conditions are fulfilled. First, the course should be offered toward the end of the curriculum, so that it offers a real, meaningful, personally important goal to the students. To first-year students, for instance, starting a career is a distant issue, and they will probably take the course primarily for its credit value. That way, due to the low level of engagement, little or no transfer happens, and the students will not have the skills and knowledge at their disposal when actually needed. Second, the course must be a curricular one, possibly with a credit value, to encourage enrollment and active participation. Third, it is important that the faculty responsible for such a course have knowledge of and be dedicated to student-centered methods. If such faculty is not available, it is worth considering the findings of Kim and Hwang [37], who identified three main characteristics of medical teachers who use student-centered methods. They found that basic sciences faculty, those with more teaching experience and those who are more knowledgeable about the school’s educational objectives show less resistance to these methods and use them more frequently. This also carries the very important message that teaching the faculty about the educational objectives of the school is a way to increase readiness to change and openness to the student-centered approach.

In summary, the success of our course shows that the often-mentioned difficulty with introducing student-centeredness in a teacher-centered environment can be overcome even in a post-socialist country where the rigidity of higher education is still a problem. The application of such methods, however, requires the careful selection of the subject, the target group and the faculty.

## Data Availability

The datasets used and/or analyzed in this study are available from the corresponding author on reasonable request.

## Abbreviations

CV: Curriculum vitae
CV/P/M: Curriculum vitae, portfolio, motivation letter
DMD: Doctor of Dental Medicine
EU: European Union
GEN: general impressions
JF: Job fair
PERS: personality and self-knowledge
PhD: Doctor of Philosophy
TECH: career-related techniques
